# Refinements in Radiography

**Published:** 1921-06-11

**Authors:** 


### WOMEN'S WORK IN THE X-RAY DEPARTMENT.

### I.—Refinements in Radiography.

BY A FEMALE RADIOGRAPHER.

Of the increasingly large number of students who have—of late years particularly—entered good hospitals for a special course of electrical and *x*-ray training, I believe by far the larger number to be women, and there is no doubt that in many ways a woman is particularly suited for this work. There are, however, at the present time a good sprinkling of incompetent "Done *x*-ray" girls and women who hold, or at least seek, positions for which they are unqualified, and thus act as a drag on the profession. The only means of doing away with this drag is to standardise the knowledge and efficiency of such students by granting them a certificate on much the same lines as that on which the diploma is now granted to medical *x*-ray specialists.

I would not for one moment advocate any *x*-ray and electrical department being run without a qualified doctor attached; but I would like to see justice done to the *x*-ray and electrical operator who, after an adequate training course for which he or she has paid a premium, is efficiently organising the department for which he or she is responsible. Such a worker is constantly on the spot and therefore knows intimately the conditions under which all radiographs are taken and developed; she watches every stage of therapeutic action, and by combining study with wide personal experience daily adds to her knowledge of electricity, photography, anatomy, disease and injury, bacteriology and radio- and electro-therapy. The radiographer also should be given the chance of sitting for a suitable examination, the passing of which will confer a definite status.

No student will ever be on the right road until she realises that even if she gives all her life to the study of *x*-ray and electrical work, yet will there always remain for her to learn far more than ever she has learnt, and that this would be true even without the constant fresh discoveries and developments which form the progress of our comparatively young but quickly growing science. She should realise also that for such knowledge as she possesses, or will ever possess, she has to thank the brains which for years past (sometimes at much cost and sacrifice to their owners) have been devoted to research work, and also the brains of to-day which are still carrying on the good work.

Not long ago I heard it said that there lay but little skill in taking radiographs, but great skill in reading them. This is quite an erroneous idea, and the outcome of ignorance in both "taking" and "reading." Truly anybody—given the apparatus in working order—can with very little instruction take radiographs of sorts; but not the best plate-reader in the world can make a difficult diagnosis from an inferior plate. On the contrary, the more skilled the reader the better plate does he demand. Moreover, though you can be an excellent plate-interpreter without being able to take radiographs, you cannot be a successful radiographer without having reached a certain point of efficiency in the matter of plate-reading, for it is more than anything else the difficulties of plate-reading which teach the best arrangement of the various parts to be radiographed in different circumstances, and also the best conditions for the development of the plates. Plate-reading also teaches the importance of accurate exposure and of regulating the tube to the condition best adapted to the particular needs of one radiograph, which vary from the particular needs of another. The mere turning on of the right switches does not take the radiograph, and the person who is regulating and choosing the tubes, organising the dark-room, requisitioning (and fighting for) the needful, making the best of the apparatus at her disposal, and, in fact, "keeping the department going," is doing work which calls for a high state of efficiency, and at the same time gives constant opportunity for further study in all directions. Almost anyone with almost any tube can demonstrate on the radiographic plate a fracture with perhaps marked displacement of fragments; but it is when soft detail in bone and the surrounding tissue is wanted, when kidney or bladder for calculi is to be examined, when a mastoid or any other intricate skull picture is required, when the first early signs of tuberculosis are looked for, or even when a child's little green-stick fracture is to be shown, that skill steps in and makes of radiography an art.

